# A Neuromotor to Acoustical Jaw-Tongue Projection Model With Application in Parkinson’s Disease Hypokinetic Dysarthria

**DOI:** 10.3389/fnhum.2021.622825

**Published:** 2021-03-15

**Authors:** Andrés Gómez, Pedro Gómez, Daniel Palacios, Victoria Rodellar, Víctor Nieto, Agustín Álvarez, Athanasios Tsanas

**Affiliations:** ^1^Old Medical School, Medical School, Usher Institute, University of Edinburgh, Edinburgh, United Kingdom; ^2^NeuSpeLab, Center for Biomedical Technology, Universidad Politécnica de Madrid, Madrid, Spain; ^3^Escuela Técnica Superior de Ingeniería Informática–Universidad Rey Juan Carlos, Móstoles, Spain

**Keywords:** speech kinematics, surface electromyography, neuromechanics, hypokinetic dysarthria, neuromotor disorders

## Abstract

**Aim:**

The present work proposes the study of the neuromotor activity of the masseter-jaw-tongue articulation during diadochokinetic exercising to establish functional statistical relationships between surface Electromyography (sEMG), 3D Accelerometry (3DAcc), and acoustic features extracted from the speech signal, with the aim of characterizing Hypokinetic Dysarthria (HD). A database of multi-trait signals of recordings from an age-matched control and PD participants are used in the experimental study.

**Hypothesis:**

The main assumption is that information between sEMG and 3D acceleration, and acoustic features may be quantified using linear regression methods.

**Methods:**

Recordings from a cohort of eight age-matched control participants (4 males, 4 females) and eight PD participants (4 males, 4 females) were collected during the utterance of a diadochokinetic exercise (the fast repetition of diphthong [aI]). The dynamic and acoustic absolute kinematic velocities produced during the exercises were estimated by acoustic filter inversion and numerical integration and differentiation of the speech signal. The amplitude distributions of the absolute kinematic and acoustic velocities (AKV and AFV) are estimated to allow comparisons in terms of Mutual Information.

**Results:**

The regression results show the relationships between sEMG and dynamic and acoustic estimates. The projection methodology may help in understanding the basic neuromotor muscle activity regarding neurodegenerative speech in remote monitoring neuromotor and neurocognitive diseases using speech as the vehicular tool, and in the study of other speech-related disorders. The study also showed strong and significant cross-correlations between articulation kinematics, both for the control and the PD cohorts. The absolute kinematic variables presents an observable difference for the PD participants compared to the control group.

**Conclusion:**

Kinematic distributions derived from acoustic analysis may be useful biomarkers toward characterizing HD in neuromotor disorders providing new insights into PD.

## Introduction

### Background

Speech production is a dynamic neuromechanical activity which involves cognitive and neuromotor resources of extreme complexity, and which is not yet well understood (Duffy, 2013). The natural way in which it is acquired and used shades the sophisticated processes which are placed into work during its normal expression. Speech is instantiated in the linguistic neuromotor cortex (Demonet et al., 2005), and its execution demands the concourse of cognitive, neuromotor, neuromuscular, and musculoskeletal processes (Duffy, 2013). Through speech, thoughts and emotions are projected to the knowledge of others by cognitive-linguistic messages. These are programmed for their neuromotor expression by the activation, time-alignment and sensorimotor projection, extension, and strength of a large amount of diverse muscles and associated biomechanical structures. The neuromotor areas from the Central Nervous System (CNS), where planning, programming and control is provided are responsible of activating the respiratory, phonatory and articulatory muscular structures innervated by the Peripheral Nervous System (PNS), see Kandel et al. (2013). The resulting speech is a sequence of acoustic interactions between the glottal source signal and the vocal tract cavities, both driven by neuromotor impulses. This imprint conveys the cognitive-linguistic message. The alteration or dysfunction of any key vocal production mechanisms will result in a deficient production of speech known as a speech disorder. Among them, Motor Speech Disorders (MSD) are the result of dysfunctional neurologic structures involved in the planning, sequencing, activating, and monitoring the neuromuscular structures responsible of speech sound production, modulation and projection. One of the most active neuromuscular structures involved in speech production is the masseter-jaw-tongue complex, including part of the facial muscles and tissues attached to the mandible (Duffy, 2013). This structure is responsible for the production of open or closed, and front or back phonations perceptible in vowels and vowel-related sounds (Greenberg, 2004). Specifically, the quasi-steady positioning of this structure (for more than 30–50 ms) gives rise to vowel-like phonations, whereas its rapid movement is responsible for the acoustical representation of many consonant-like sounds. Neuromotor diseases affect the functional operation of this structure, and its central role in speech articulation suggests it could likely reflect key pathological changes reflected the neuromotor behavior. A well-known indicative neuromotor disorder known for its prevalence and social impact is Parkinson’s Disease (PD), also known as shaking palsy. It is a well-known neurodegenerative disorder since it was first described by J. Parkinson (2002). Its etiology is unclear in most of the cases, but evidence is accumulated in the sense that it may be due to different dysfunctions taking place in the fine control of muscular actions in the interplay of the brain subsystems responsible of musculoskeletal control, as the hypothalamus, the cerebellum, the primary and secondary neuromotor control areas, and the frontal lobes (Brown et al., 2009). A compelling and comprehensive overall view is given in Duffy (2013): “*The motor system is present at all of the major anatomic levels of the nervous system and is directly responsible for all motor activity involving … to the planning, control, and execution of voluntary movement, including speech.*” It is a well-established fact that PD causes considerable alterations in speech and phonation (Ricciardi et al., 2016; Brabenec et al., 2017). Broadly speaking, speech alterations may be classified as dysphonia (on voice production), dysarthria (on speech articulation), dysprosody (on the fundamental frequency sequence), and dysfluency (on the rhythm and speech sequence of intersyllabic and intersegment blocks). These alterations are jointly referred to as Hypokinetic Dysarthria (HD). Harel B. et al. (2004) give a summary of the symptoms associated with HD “*Hypokinetic dysarthria, a speech disorder characterized by indistinctness of articulation, weakness of voice, lack of inflection, burst of speech, and hesitations and stoppages, is an integral part of the motoric changes in PD.*” In this same sense, there is “*compelling evidence to suggest that speech can help quantify not only motor symptoms… but generalized diverse symptoms in PD*” (Tsanas, 2012). Godino et al. (2017) stress the fact that “*The low levels of dopamine that appear in patients with PD lead to dysfunctions of the basal ganglia… These deficits negatively affect the three main anatomic subsystems involved in the speech production: respiration, phonation and articulation.*” A good description of the neuromotor systems involved in speech production, and how they may be affected by neurodegenerative diseases is to be found in Duffy (2013). Therefore, the search of neuromotor degenerative biomarkers in speech is to be concentrated on phonation (study of the glottal signals in terms of distortion and biomechanics), on speech articulation (study of acoustic and biomechanic clues as formants and jaw-tongue kinematics), on the prosodic flow (concentrated in the time evolution of the fundamental frequency and speech energy stability), and on fluency (syllabic and intersyllabic intervals, duration, stability, and fluctuation of the speaking rate). This is well documented in the work of Mekyska et al. (2015). Moreover, speech can be used to investigate the nature and extent of vocal impairment in individuals who are at risk of developing PD and can provide a crucial opportunity to intervene in prodromal stages. For example, we have recently demonstrated some very compelling findings when comparing speech signals between a control group, people diagnosed with Sleep Behavior Disorder (SBD), which is among the strongest known predictors of PD risk, and a PD cohort (Arora et al., 2021). Furthermore, we have demonstrated that we can accurately telemonitor PD symptom severity using speech signals collected over the standard telephone network, thus alleviating the need for frequent physical patient visits to the clinic (Tsanas et al., 2021). The main compelling facts favoring speech-based PD biomarkers are the low cost of the required equipment, smart-phones and tablets being increasingly affordable and generally accessible devices, and the contact-less factor, which is particularly useful to facilitate remote studies. Summarizing, the acoustic markers induced by HD in PD speech allows to conclude that speech analysis might become a non-invasive and cost-effective tool to characterize and monitor PD. The role of speech as a possible biomarker in PD detection is well established in the state of the art research literature, with many studies discussing speech-based PD features sensitive to HD. In the present study the focus is placed on the study of acoustic and biomechanical clues, as formants, and jaw-tongue kinematics.

### Previous Work

The number of studies focusing on the field of speech and neurodegenerative disorders has been consistently growing over the last 10–15 years, and for the sake of brevity only the most relevant ones will be mentioned in what follows. Brown et al. (2009) give a good description of the relationship between the premotor and motor cortex areas with speech production. Good descriptions of the neuromotor system of the phonation and articulation including neuromotor to muscular pathways affecting the larynx, pharynx, oral, and nasal cavities may be found in Jürgens (2002). Bourchard et al. (2016) offer a description of the relationship between simultaneously recorded neural activity and the kinematics of the lips, jaw, tongue, and larynx. An interesting review on neurophysiology of language may be found in Demonet et al. (2005). Relevant information on the neuromotor pathways on vocal control can be found in Goodman and Hasson (2017). A classical and detailed biomechanical description of tongue movement control may be found in Sanguinetti et al. (1997). The influence of PD on facial muscle sEMG is given in Wu et al. (2014), and for the articulatory and acoustic changes due to Amyotrophic Lateral Sclerosis (ALS) see Mefferd and Dietrich (2020). Relating acoustic features to articulation gesture the reader may see, Gerard et al. (2006), Buchaillard et al. (2009); Dromey et al. (2013). On diphthong articulation kinematics see Tasko and Greilick (2010); for tongue positioning during vowel articulation in speakers with dysartria see Yunusova et al. (2011); for tongue movements in speakers with ALS see Yunusova et al. (2012). For the use of speech signals from longitudinal assessment of PD we refer to Tsanas et al. (2011) and Tsanas (2012), and the specific ones by Green et al. (2013); Sapir (2014), Mekyska et al. (2015), or Brabenec et al. (2017). On PD and multiple sclerosis see Tjaden et al. (2013), for vowel articulation positions as a marker of neurodegenerative progress see Skodda et al. (2012).

### Objectives

The present study builds on previous work to characterize the relationship between acoustic, 3D accelerometry traces, and electromyographical correlates of speech, in relation with the jaw-tongue structure when carrying on diadochokinetic exercises of clinical interest in neuromotor degenerative disorders as PD (Gómez et al., 2019a,b). We have three primary objectives in the study. Firstly, it is focused to evaluate the functional relationship between neuromotor action in the masseter derived from sEMG and 3Dacc with respect to the acoustic outcomes measured by the two first formants on signals produced both by male and female controls and PD participants exercising on a specific voiced diadochokinetic utterance (repeated sequence of [aI], according to the International Phonetic Alphabet, IPA, 2005). It is assumed that cross-correlations between sEMG, 3D acceleration, and acoustic features may help in determining the optimal time-delays to estimate the weights of projection models by linear regression methods. Secondly, we aim to explore the possibility of establishing inverse relationships between acoustic features and neuromotor activity estimated from sEMG measured on the masseter. Thirdly, we aim to establish whether there are notable differences between controls and PD participants in terms of the absolute kinematic behavior derived from the diadochokinetic exercise from the repetition of [aI] to define new HD biomarkers.

## Materials and Methods

### Speech Neuromechanics

**FIGURE 1:**
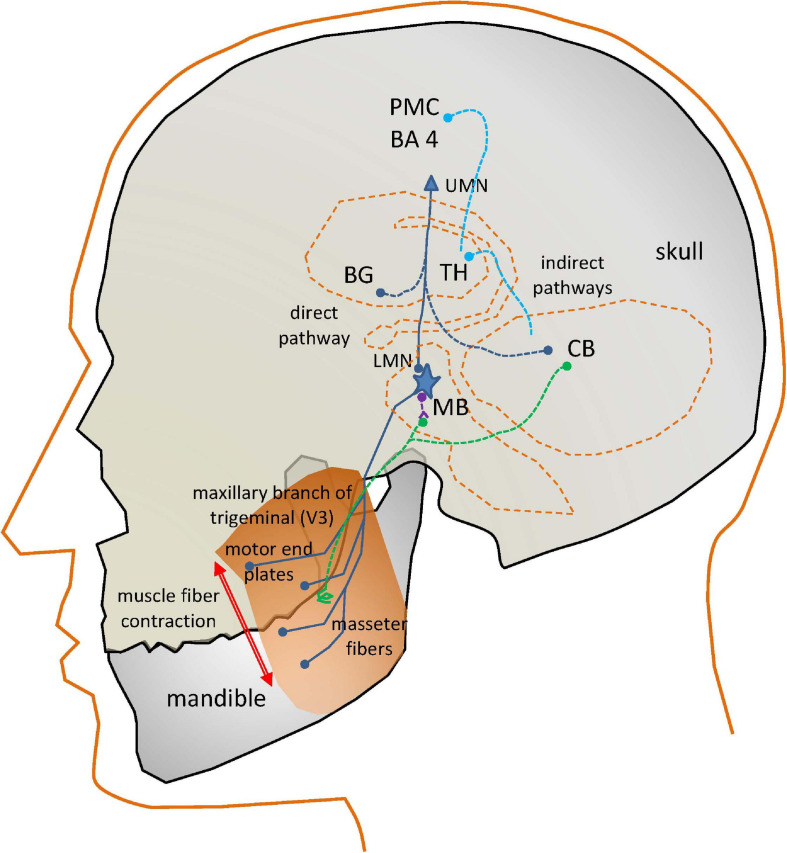
Simplified view of the neural circuits involved in the action of the masseter neuromotor units. PM: Pre-motor Cortex (BA4, Brodmann’s Area 4); UMN, Upper Motor Neuron; BG, Basal Ganglia; TH, Thalamus; CB, Cerebellum; LMN, Lower Motor Neuron; MB, Midbrain; dark blue, direct and final common pathways; light blue, indirect control pathways from CB and BG; green, sensory pathways; purple, inhibitory pathways.

Speech is a complex activity which involves the coordinated joint action of respiration, phonation and articulation muscles, controlled by different cranial nerves (V, VII, IX, X, and XII) and phrenic nerves from the spinal cord, which are responsible for producing speech (Duffy, 2013). Motor activity related to speech is expressed by the cortical areas for motor speech planning and programming of the Central Nervous System (CNS) in Brodmann Area 4 (Primary Motor Cortex: PMC) located in the dorsal portion of the frontal lobe (see Figure 1). The programmed activity is transferred to the Peripheral Nervous System (PMS) by connections from the PMC, working in association with other motor areas (premotor cortex, supplementary motor area, posterior parietal cortex, and other subcortical brain regions, which play a role on motor planning and execution). The Upper Motor Neurons (UMNs) are neurons in the PMC, which together with other cortical areas connect with the Lower Motor Neurons (LMNs) in the PMS by the corticobulbar and corticospinal tracts. This is known as the direct pathway. LMNs are alpha-type motor neurons whose axons directly activate the muscles. The LMN and the muscle fibers it activates is known as the Motor Unit (MU). The MUs activating a given muscle, are known as the Final Common Pathways (FCPs). In the case of the masseter (the subject of the present study), the FCPs are grouped in the trigeminal maxillary branch, the third subdivision of the cranial nerve V (V3).

In the case of interest for the present study, the motor end plates of the FCPs innervate the masseter fibers producing the muscle contraction. The masseter activation is induced as well by indirect pathways. The fine control of a muscle movement requires some kind of feedback. This is provided by sensory pathways (in green) consisting in neurons activated by spindles (terminal sensors detecting fiber stretching) attached to the muscles, providing proprioceptive sensing to the LMNs in two ways. A direct feedback loop is provided by inhibitory interneurons (in purple). A more complex feedback loop connects sensor units with the Basal Ganglia (BG) and the Cerebellum (CB). These structures serve feedback information to the motor and frontal cortices, as well as to the LMN (blue lines). The BG control circuit assists the PMC in accurate and fine motor speech programming. The CB control circuit coordinates PMC motor planning from proprioceptive information.

Motor speech disorders are produced by specific problems affecting some of the described direct or indirect pathways of activation, or the muscle fibers. These disorders are commonly referred to as dysarthrias. In the case of PD, the speech disorder is known as HD, related with the pathological behavior of the complex BG control circuit. It will affect any or all the mentioned systems responsible for the neuromechanical control of speech: respiration, phonation, and articulation. The term “hypokinetic” refers to weak small range and rigid movement, giving the impression of flat, soft, and expressionless speech (Duffy, 2013). The activity of the BG control circuit is inhibitory on the PMC areas to modulate cortical activity. An excessive inhibition may result in HD. Most motor problems related with the BG motor circuit have to see with neurotransmitters. Specifically, dopaminergic deficits due to the progressive death of dopaminergic neurons in the substantia nigra *pars compacta* (located at the MB) are the main reason behind PD neuromotor symptoms: “*The substantia nigra is the origin of the nigrostriatal pathway, which travels to various structures within the basal ganglia… The dopamine deficiency in this nigrostriatal pathway and the basal ganglia account for most of the typical features of PD. Once the brain is no longer able to compensate for this dopamine loss, there are a number of effects which can occur. Typical symptoms include muscle rigidity, akinesia, bradykinesia, and tremor…*” (Goberman and Coelho, 2002), more specifically “*The essential neuropathological changes in PD are a loss of melanine-containing dopaminergic neurons in the substantia nigra pars compacta… This results in a dysfunction of the basal ganglia circuitry, which is an integral part of cortico-basal ganglia-cortical loops that mediate motoric and cognitive functions*” (Harel B.T. et al., 2004).

In the present study, a biomechanical system of the jaw-tongue will be used, which may be modeled to estimate the neuromotor behavior of the system, and provide specific markers of proper or improper neuromotor activity. The selection of the masseter as the target muscle obey to the following reasons: it is a powerful muscle developing a strong sEMG when contracting, it is accessible (beneath the caudal section of the cheek), it may modify strongly the oral cavity when contracting or relaxing leaving a clear acoustic signature in formants, and its biomechanical activity is well understood. The study of the masseter neuromechanics is based on the electrical, dynamic, and kinematic activity of the muscle as a functional structure.

### A Model Mediating Jaw Kinematics on Acoustic Features

**FIGURE 2:**
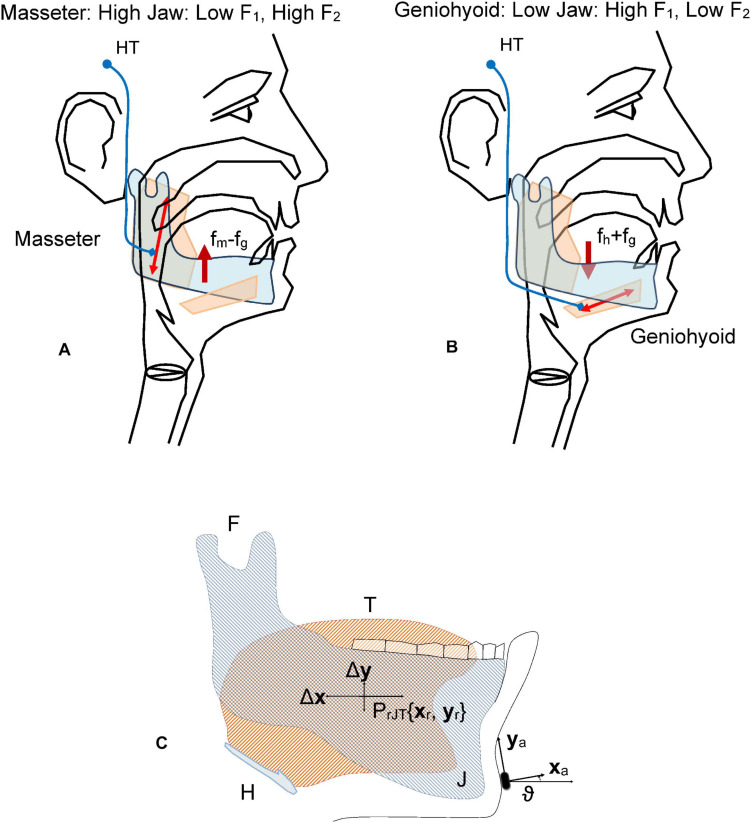
Fundamentals of the jaw-tongue articulation. **(A)** Upper pulling (levator active against gravity, depressor inactive) As the jaw is high, the first formant F1 is low. **(B)** Lower pulling (depressor and gravity active, levator inactive) As the jaw is low the first formant is high. **(C)** Jaw-Tongue Biomechanical System. The jaw bone is represented in light gray; the tongue structure is represented in light orange. The point PrJT given by {xr, yr} is the reference point of the biomechanical system. HT, Hypothalamus; fm, force exerted by the masseter; fg, gravity force; fh, force exerted by the geniohyoid; F, attach joint of the jaw to the skull; H, hyoid bone; T, tongue (in orange); J, jaw (in gray).

The present study builds on a previous masseter-jaw-tongue biomechanical model (Gómez et al., 2019b), where the active contribution of other muscles (styloglossus, geniohyoid, intrinsic glossal, etc.) has not been taken into account, and the passive contribution of other tissues, as the oro-facial substructures are implicitly included in the inertial moment. The articulation gestures based on the masseter-jaw-tongue structure as considered in the present study are described as follows: the jaw (J) is fixed to the skull bone at fulcrum (F) as in a third-class lever system. The tongue (T) is a complex muscular and vascular structure supported on the jaw and the hyoid bone. Some other extrinsic muscles fix it to the cranial structure (mainly the styloglossus and geniohyoid). These muscles and the tissues surrounding the jaw will be considered part of the lumped equivalent of masses, forces and moments at the reference point of the jaw-tongue system *P*_*rJT*_, defined at {*x*_*r*_*,y*_*r*_}, where forces acting on the system induce movements in the sagittal plane (*x*: horizontal, or rostral-caudal, *y*: vertical, or dorsal-ventral); these forces are *f*_*m*_ (exerted by the masseter), *f*_*sg*_ (by the styloglossus), *f*_*gi*_ (by the intrinsic glossus), *f*_*h*_ (by the geniohyoid), and *f*_*g*_ (gravity). Consequently, the *P*_*rJT*_ may be defined as a hypothetical point in the sagittal plane (*x*: caudal-rostral; *y*: dorsal-ventral) with static coordinates {*x*_*r*_, *y*_*r*_} where the sum of dynamic and inertial forces is null. The force exerted by the masseter *f*_*m*_ will pull up the low mandible acting as a third-order lever with fulcrum at the maxillary attachment (F). In the present study we will not consider extrinsic actions other than by geniohyoid, and by the intrinsic glossus system, therefore, the only forces to be considered contributing to the lever momentum will be *f*_*m*_, *f*_*h*_, and *f*_*g*_. The kinematic displacements experienced by *P*_*rJT*_ are given as {Δ*x*_*r*_Δ*y*_*r*_}. Lateral movements orthogonal to the sagittal plane are assumed small enough not to be considered (system with only two degrees of freedom). The functioning of the speech articulation neuromotor and biomechanical system is ilustrated Figure 2. Speech articulation is defined by the configuration of the articulation organs, such as the mandible, tongue, lips, and velo-pharyngeal tissues, among others of lesser interest for the present study (Dromey et al., 2013; Cattaneo and Pavesi, 2014; Whitfield and Goberman, 2014). Therefore, vowel articulation has been traditionally described in terms of the open-close gesture (also low-high attending to lower jaw position relative to upper jaw), the front-back gesture, and round-oval configurations (Dromey et al., 2013). These gestures determine certain acoustic features known as formants, which are defined as frequency positions where the harmonic structure of the vowel is especially enhanced, by means of the resonances of the Oro-Naso-Pharyngeal Tract (ONPT, see Figure 2). The open-close gesture, mainly dominated by the jaw, is affecting the first formant *F*_1_. Pulling up the jaw by the masseter against gravity (*f*_*m*_−*f*_*g*_) is the dominant gesture in the phonation of high vowels as [i:] and [u:], whereas depressing the jaw under the force of gravity and the geniohyoid muscle action (*f*_*h*_+*f*_*g*_) is the gesture to phonate low vowel as [a:]. The front-back gesture is controlled also by the jaw position, although in high vowels like [i:] and [u:] the tongue position affects mainly the second formant *F*_2_ (pushing the tongue forward is the gesture for [i:], pulling it back results in [u:]). Therefore, the articulation gesture of the jaw may be studied to relate articulatory gestures and acoustic features as the first two formants (*F*_1_, *F*_2_), as proposed in the Articulation Kinematic Model (AKM) shown in Figure 2C. The study is based on the dynamic tracking of the kinematic activity of the jaw-tongue reference point (*P*_*rJT*_) by means of 3D accelerometry (3Dacc).

The articulation dynamics foresees that the resonant properties of the ONPT will change in time regarding the position of the *P*_*rJT*_ under the action of the forces mentioned, modifying, and producing dynamic changes in the first two formants{*F*_1_, *F*_2_}. From the perspective of speech production, the general problem of acoustic to articulatory inversion may be presented in general terms as the mapping A(**s**) = **F** (Ouni and Laprie, 2005), where **s** is the articulatory vector which presents the general shape of the vocal tract at a specific time instant for instance, the *k* parameters of an articulatory synthesis model, and **F** is the acoustics vector (typically the first m formants). A: **s**→**g** is a non-linear many-to-one mapping transforming the articulatory feature space **s** to the acoustic feature space **g**. In this sense, the inverse mapping presents several important problems: on the one hand the mapping A is non-linear, on the other hand, it is a many-to-one (Qin and Carreira-Perpiñán, 2007). Besides, **g** is an approximation to the values of the true resonances of the vocal tract, obtained usually from Linear Prediction Inverse Filtering (LPIF), and therefore, subject to strong assumptions on the representation of the real oro-naso-pharyngeal tract (ONPT) by a concatenation of rigid-wall cylindrical-tube sections on a straightened medial axis, the number of sections, depending on the sampling rate and the order of the LPIF (Deller et al., 1993). On the other hand, the feature vector **s** will depend on the specific generative model used. In many studies the seven following features are used: jaw position (high-low), tongue dorsum position (backward-forward), tongue dorsum shape (rounded-unrounded), tongue tip shape (up-down), lip height (open-close), lip protrusion (forward-backward), and larynx height (high-low). The solutions proposed are based on reproducing a reduced articulatory feature space **s** which may generate an approximation to the acoustic feature vector **g**, following an optimization process reducing the cost function | **g**-**g**| to a minimum value. Several approaches are used such as codebook-based inversion, articulatory modeling, articulatory modeling, and statistical mapping (Sivaraman et al., 2019). The present study proposes a mapping model which is a simplification of the general one following several important assumptions:

• Only the vertical and horizontal components of the jaw-tongue position (high-low) are considered from the set of articulatory features s. These features are estimated using a 3D accelerometer fixed to the participant’s chin. A transformation of coordinates from the 3D accelerometer to absolute ones in the sagittal plane is used (rotation and projection).
• The set of acoustic features is reduced to the first two formants *F*_1_ and *F*_2_.
• A kinematic-acoustic variable estimated from formant acoustics (AFV).
• A kinematic-dynamic variable estimated from jaw kinematics (AKV).
• A non-linear model projecting acoustic features to a kinematic-acoustic variable is proposed
• A linear model mapping acoustic features to dynamic variables is proposed.

Having these assumptions in mind, the present study is focused on the evaluation of a linear model on data produced by control and PD participants in the fast and repeated utterance of the diphthong [aI]. The reasons for selecting such a diadochokinetic exercise are that, on the one hand this pattern ensures that the jaw-tongue system moves through a range where linearity governs the link between articulation and sound features “*For /aI/ the strength of the acoustic-kinematic association was robust across movements that differed in duration or displacements*” (Dromey et al., 2013). On the other hand, this exercise is mainly governed by the dynamic activity of the masseter, a very accessible facial muscle producing good sEMG recordings. Another important fact is that this exercise does not involve lip protrusion or rounding changes (Dromey et al., 2013). Therefore, the association between the jaw-tongue reference point movement may be associated with the first two formants in a one-to-one mapping. The complementary use of a 3D accelerometer on the jaw helps in providing a second assessment method to avail this association by an alternative acoustic-to-dynamic mapping.

The proposed model for projecting articulation kinematics to changes of the first two formants *F*_1_ and *F*_2_ can be described as:

(1) $$\left[\begin{array}{c} △\,F_{1}\,\left(t\right)\\ △\,F_{2}\,\left(t\right) \end{array}\right]=\left[\begin{array}{cc} a_{11} & a_{12}\\ a_{21} & a_{22} \end{array}\right]\,\left[\begin{array}{c} △\,x_{\text{r}}\,\left(t\right)\\ △\,y_{\text{r}}\,\left(t\right) \end{array}\right]$$

where {*a*_*ij*_} are the parameters relating the dynamic changes of the formant values with the oscillations of the *P*_*rJT*_. The utility of these relations is conditioned by the possibility of estimating the set of parameters {*a*_*ij*_} from the signals produced by a 3D accelerometer fixed on the chin of a participant under test, as shown in Figure 2C. The accelerometer reference axes are the chin-normal (***x***_*a*_) and tangential (***y***_*a*_), which will be changing following jaw displacements. The accelerometry signals may be transformed to the reference coordinates {*x*_*r*_, *y*_*r*_} by means of a rotation in terms of *ϑ*, the angle between the axes ***x***_*a*_ and ***x***_*r*_. The set of parameters {*a*_*ij*_} relating acoustic features and articulation dynamics may be estimated by regression-based methods using specific repetitive diadochokinetic exercises, such as the repetition of the gliding sequence […aIa*…*] as it will be shown in what follows. Assuming that (1) is time-invariant and invertible we would have:

(2) $$\left[\begin{array}{c} d\,x_{\text{r}}\,\left(t\right)/d\,t\\ d\,y_{\text{r}}\,\left(t\right)/d\,t \end{array}\right]=\left[\begin{array}{cc} w_{11} & w_{12}\\ w_{21} & w_{22} \end{array}\right]\,\left[\begin{array}{c} \partial F_{1}\,\left(t\right)/\partial t\\ \partial F_{2}\,\left(t\right)/\partial t \end{array}\right]$$

where {*w*_*ij*_} are the coefficients of the transference matrix inverse ***W*** = ***A***^–1^:

(3) $$\left[\begin{array}{cc} w_{11} & w_{12}\\ w_{21} & w_{22} \end{array}\right]={\left[\begin{array}{cc} a_{11} & a_{12}\\ a_{21} & a_{22} \end{array}\right]}^{-1}$$

With these expressions in mind it will be possible to define the Absolute Formant Velocity (AFV) of the reference point (*P*_*rJT*_) as:

(4) $$\left|v_{\text{f}}\,\left(t\right)\right|={\left[H_{1}\,{\left(\frac{d\,F_{1}\,\left(t\right)}{d\,t}\right)}^{2}+H_{2}\,{\left(\frac{d\,F_{2}\,\left(t\right)}{d\,t}\right)}^{2}+H_{12}\,\frac{d\,F_{1}\,\left(t\right)}{d\,t}\,\frac{d\,F_{2}\,\left(t\right)}{d\,t}\right]}^{1/2}$$

where *H*_1_, *H*_2_ and *H*_12_ are quadratic forms of {*w*_*ij*_} (see Gómez et al., 2019b). Reliable estimates for {*w*_*ij*_} may be obtained from articulations involving changes in the positions of the reference point showing predictable dynamic changes, as in this and other diadochokinetic exercises. The AFV may be estimated in the following steps:

• Speech *s*(*t*) is sampled at 50 kHz and 16 bits, down-sampled to 8 kHz and inverse filtered (Alku et al., 2019) to obtain a K-th order adaptive prediction vector {*b*_*i*_} representing the inverse vocal tract.
• The first two formants *F*_1_ and *F*_2_ are estimated by detecting the zeros of *B*(*z*) in the complex plane:

$$B\left(z=z_{\text{i}}\right)=1-\sum_{\text{i}=1}^{\text{k}}b_{\text{i}}z_{\text{i}}^{-\text{i}}=0;$$

(5) $$z_{\text{i}}=r_{\text{i}}\,e^{j\,\varphi_{\text{i}}};F_{\text{i}}=\varphi_{\text{i}}/\left(2\,\pi \,\tau \right);\varphi_{\text{i}}\geq 0$$

τ being the sampling rate in the time domain.

Similarly, an Absolute Kinematic Velocity (AKV) of the reference point may be derived from the normal and tangential acceleration components on the sagittal plane {*a*_*Xa*_(*t*), *a*_*Ya*_(*t*)} measured directly by the 3D accelerometer as:

(6) $$\left|v_{\text{d}}\,\left(t\right)\right|={\left[{\left(\int_{\zeta =0}^{t}a_{\text{xd}}\,\left(\zeta \right)\,d\zeta \right)}^{2}+{\left(\int_{\zeta =0}^{t}a_{\text{yd}}\,\left(\zeta \right)\,d\zeta \right)}^{2}\right]}^{1/2}$$

where {*a*_*x*__*d*_(*t*), *a*_*y*__*d*_(*t*)} are the acceleration components in the sagittal plane rotated from the measurements recorded on the native accelerometer axes {*a*_*Xa*_(*t*), *a*_*Ya*_(*t*)}.

The estimation procedure of the AKV would involve the following steps:

• The acceleration component means {*â*_*Xa*_(*t,W*), *â*_*Ya*_(*t,W*)} are used to estimate the 3D accelerometer angle *ϑ* on short time windows (*W*) to preserve time invariance. The acceleration components are rotated to the reference axes to produce {*a*_*x*__*d*_(*t*), *a*_*y*__*d*_(*t*)}.
• Rotated acceleration components are used to estimate the AKV following (6).

### Distributions of Absolute Velocity Values

The probability distributions of both AFV and AKV from (4) and (6) are very relevant statistical descriptors of articulation kinematics. They can be directly estimated from their normalized amplitude histograms over bins between 0 and 20 (cm.s^–1^) as:

• The speech signal is low-pass filtered to 4 kHz (antialiasing) and downsampled to 8 kHz. The 3D acceleration signals are low-pass filtered to 250 Hz and downsampled to 500 Hz.
• The first two formants are estimated every 2 ms on short time windows of 64 ms, equivalent to 512 samples with an overlap of 97% (62/64).
• The weights {*w*_*ij*_} are estimated from linear regression between dynamic displacements {Δ*x*_*r*_, Δ*y*_*r*_} and formant deviations {Δ*F*_1_, Δ*F*_2_}. The quadratic coefficients *H*_1_, *H*_2_, and *H*_12_ are calculated from {*w*_*ij*_}.
• The AFV (| *v*_*f*_(*t*)|) is estimated from (4).
• The accelerations {*a*_*x*__*d*_(*t*), *a*_*y*__*d*_(*t*)} are obtained by rotating the signals recorded by the 3D accelerometer downsampled to 500 Hz and integrated numerically. Detrend filtering must be used to avoid integration drifts.
• The AKV (| *v*_*d*_(*t*)|) is estimated from (6).
• An N-bin histogram of counts by amplitudes is built from each participant’s AFV and AKV. The interval covered for speeds is [0, | *v*_*r*_| _*max*_], with | *v*_*r*_| _*max*_ = 20 cm.s^–1^, and *N* = 100, therefore each bin size is Δ*b*_*k*_ = [| *v*_*r*_| _*max*_/N] = 0.2 cm.s^–1^ wide.
• The following histogram of counts is built for each bin *b*_*k*_ = *k*•Δ*b*_*k*_:
  *if b*_*k*__–__1_ ≤ | *v*_*r*_(*t*)| *< b*_*k*_ *then c*_*k*_ = *c*_*k*__+__1_
  *c*_*k*_ being the number of counts for bin *b*_*k*_.
• Count histograms *c*_*k*_ (0 ≤ *k* ≤ N) are normalized to their total number of counts *C*_*T*_ = Σ*b*_*k*_ (0 ≤ *k* ≤ N), therefore they could be considered estimators of probability density functions *p*_*k*_ = *c*_*k*_/*C*_*T*_.

Thence *p*(| *v*_*rk*_|) = *p*_*k*_ will be an estimate of the AFV and AKV probability density functions. It has been proven that this feature is relevant in separating dysarthric from normative speech (Gómez et al., 2017).

### Entropy-Based Detection

The AFV and AKV distribution show a two-degrees of freedom χ^2^ behavior, therefore they are an estimation of the speaker’s jaw mobility in terms of similarity with a certain “articulation temperature.” The probability distributions may be used to estimate the divergence between utterances from different speakers in terms of entropy based metrics (Cover and Thomas, 2006) between two probability distributions (i, j) in terms Jensen-Shannon’s Divergence (JSD) as:

$$D_{\text{JSij}}\,\left\{p_{\text{i}}\,\left(\left|v_{\text{r}}\right|\right),p_{\text{j}}\,\left(\left|v_{\text{r}}\right|\right)\right\}=\frac{1}{2}\,D_{\text{KL}}\,\left\{p_{\text{i}},p_{\text{m}}\right\}+\frac{1}{2}\,D_{\text{KL}}\,\left\{p_{\text{j}},p_{\text{m}}\right\};$$

(7) $$p_{\text{m}}=\frac{p_{\text{i}}+p_{\text{j}}}{2}$$

where *p*_*i*_(*v*_*r*_) and *p*_*j*_(*v*_*r*_) are two distributions (either AFV or AKV) from two different participants, and *D*_*KL*_ is Kullback-Leibler’s Divergence (Cover and Thomas, 2006):

(8) $$D_{\text{KLij}}=D_{\text{KL}}\,\left\{p_{\text{i}}\,\left(\left|v_{\text{r}}\right|\right),p_{\text{j}}\,\left(\left|v_{\text{r}}\right|\right)\right\}=-\int_{\zeta =0}^{\infty }p_{\text{i}}\,\left(\zeta \right)\,l\,o\,g\,\left[\frac{p_{\text{i}}\,\left(\zeta \right)}{p_{j}\,\left(\zeta \right)}\right]\,d\zeta$$

The main advantage of JSD is that it is bounded and symmetrical (*D*_*JSij*_ = *D*_*JSji*_). The AFV and AKV estimates from (4) and (6) and their normalized histograms were evaluated as described before. Two sets of distributions were produced, respectively, for the control participants (CP: *p*_*CP*_) and the PD participants (PD: *p*_*PP*_}. The average of the p_*CP*_ distributions was used as the control reference:

(9) $$p_{\text{CP}}=\frac{1}{k_{\text{C}}}\,\sum_{\text{i}=1}^{k_{\text{C}}}p_{\text{CPi}}$$

where *k*_*C*_ is the number of participants in the CP set separately for AFV and for AKV. The JSD between each participant in the sets CP and PD was estimated with respect to the average *p*_*CP*_. Therefore, the divergences used in the present study are:

$$D_{{\text{JSf}}_{\text{i}\in \text{CP}}}=D_{\text{JS}}\,\left\{p_{\text{if}},p_{\text{CPf}}\right\};D_{{\text{JSf}}_{\text{i}\in \text{PD}}}=D_{\text{JS}}\,\left\{p_{\text{if}},p_{\text{CPf}}\right\};$$

(10) $$D_{{\text{JSd}}_{\text{i}\in \text{CP}}}=D_{\text{JS}}\,\left\{p_{\text{id}},p_{\text{CPd}}\right\};D_{{\text{JSd}}_{\text{i}\in \text{PD}}}=D_{\text{JS}}\,\left\{p_{\text{id}},p_{\text{CPd}}\right\}$$

where the suffixes *f* and *d* specify JSDs estimated from AFV or AKV, respectively.

### Masseter sEMG

The selection of the masseter as the reference muscle in studying the relationship of neuromotor activity and acoustic speech dynamics is based on the following reasons:

• The masseter changes the position of the jaw-tongue structure toward high and slightly forward positions when activated. Correspondingly, the vocal tract is modified from low-mid to high-front vocal resonances (Dromey et al., 2013). The relationship between neuromuscular action and acoustics seems to be quite direct.
• The masseter is a powerful muscle, its neuromotor activity induces strong sEMG signals on the back lower part of the cheek. Signal recording is feasible and very productive.
• The neuromotor pathways from the midbrain to the masseter are short and fast, the delays due to impulse propagation on the neural pathways, and the motor unit action potentials are small. This allows producing faster oscillation movements than other larger and more distant muscles, extending the high frequency limits of voluntary and involuntary tremor.

**FIGURE 3:**
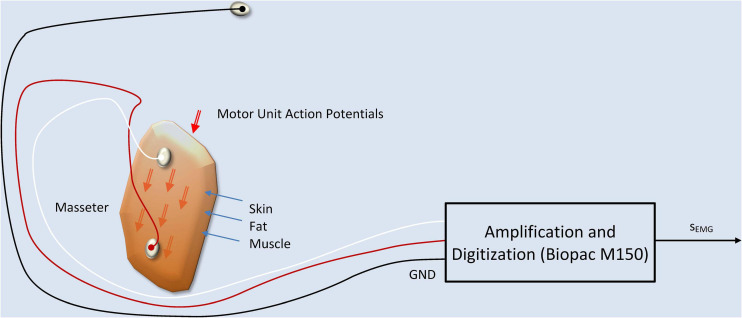
sEMG production and recording model. The two active electrodes are fixed at both ends of the masseter muscle, on the skin, capturing a signal which is the additive composition of the Motor Unit Action Potentials (MUAPs) traveling along the muscle fibers. A ground electrode fixed at the forehead serves as a reference point. The equipment used in the recordings is a WiFi-connected electrode terminal unit which communicates the signals on a wireless link to a Biopac M150 recording system.

The procedure to record sEMG activity on the masseter is represented in Figure 3 following the works of De Luca (1997) and Castroflorio et al. (2005).

The sEMG signal recorded is related with the summation of the MUAPs traveling along the muscle fibers on the muscle cell membranes as described in Martínez-Valdés et al. (2017):

(11) $$s_{\text{EMG}}\,\left(n\right)=\sum_{k}H\,\left(k\right)\,s_{\text{MUAP}}\,\left(n-k\right)\,e\,\left(n\right)$$

where *s*_*EMG*_(n) is the recorded sEMG signal, *H*(*k*) is a transfer weight having into account skin and fat conductivity and time propagation effects, *s*_*MUAP*_(*n*) are the MUAPs traveling along the muscle fibers, *e*(*n*) is the recording noise, and *n* is the time index (Farina and Merletti, 2001; Teklemariam et al., 2016). Concerning the dynamic action exerted by the muscle during activation it will be assumed that the force exerted by the muscle in the upper vertical direction *f*_*m*_(*t*) is a correlate of the sEMG recorded on the bulk of the masseter *s*_*m*_(*t*) (Roark et al., 2002; Lee, 2010) and therefore, it may be expressed as the joint action of these individual actions, therefore:

(12) $$f_{\text{m}}\,\left(t\right)=J_{\text{m}}\,r_{\text{m}}\,\left(t\right);J_{\text{m}}=\frac{T_{\text{m}}}{l_{\text{m}}\,c\,o\,s\,\vartheta };r_{\text{m}}\,\left(t\right)=\int_{\zeta =0}^{t}\left|s_{\text{m}}\,\left(\zeta \right)\right|\,d\zeta$$

where *J*_*m*_ is the mioelectric proportionality parameter when small oscillations are assumed, *T*_*m*_ is the angular neuromotor torque, *l*_*m*_ is the effective jaw arm length (considering the jaw-tongue system as a lumped load), *ϑ* is the rotation angle (Hogan, 1984) and *r*_*m*_(*t*) is the integral of the rectified sEMG on the masseter. The sEMG is low-pass filtered at 250 Hz and downsampled to 500 Hz. Its rectified value is numerically integrated following (12). Detrending filtering must be used to avoid integration drifts. The reason behind force being related to the integral of the rectified sEMG, and not to sEMG, has to see with the way in which sEMG is recorded, using pairs of electrodes symmetrically placed at both sides of the neuromotor innervation zone on the muscle (see Figure 3), as suggested by the experts (De Luca, 1997; De Luca et al., 2010). The integral of the rectified sEMG will be referred in the sequel as the Electromyographic Correlate of the Masseter Force (ECMF).

### Linear Regression-Based Statistical Mapping

The present study is intended to link the masseter neuromotor activity (estimated from the sEMG signal) with jaw-tongue kinematics (estimated from 3D accelerometry) and acoustic dynamics (estimated from the first two formants of voice). Cross-correlation (time lag correlation) is used to estimate the optimum time-lag alignment between two signals, determined by the maximum absolute values of Pearson’s correlation coefficients. Linear regression between the aligned signals is used to estimate the weights of the corresponding projection model. Cross-correlation methods have been already used in acoustic-kinematic mapping are standard procedures (see Ouni and Laprie, 2005; Dromey et al., 2013; Mitra et al., 2017; Sivaraman et al., 2019). The aim of the present study is based on the inherent relationship between the masseter activity and jaw movement, to show that the front-to-end causality chain from Neuromotor Activity → Masseter sEMG → Vertical Force → Vertical Position → {ΔF1, ΔF2} may be quantitatively described by a simple model, and that the model parameters might be estimated in a first approximation by linear methods. Having in mind that the force exerted by the masseter could not be inferred directly, the present study opens the possibility of making this estimation possible relating acoustic features with neuromotor activity in an inverse relationship, using speech, sEMG and 3D accelerometry.

**FIGURE 4:**
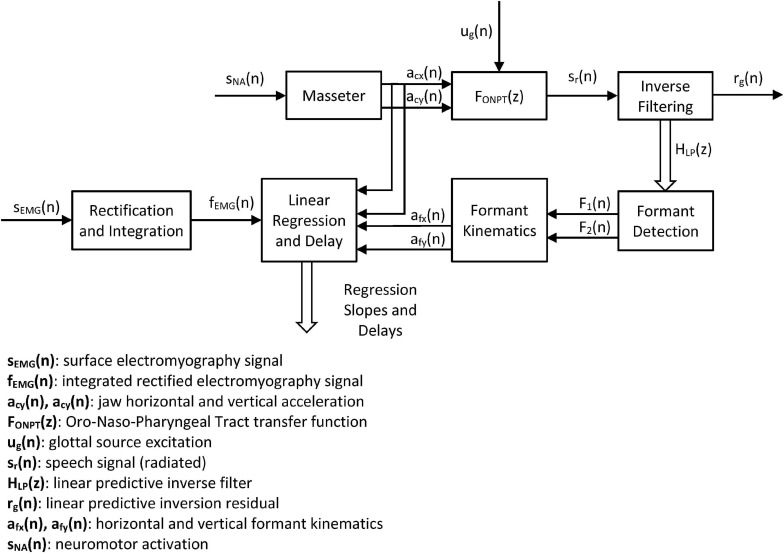
Systemic view of the study. The neuromotor activity induced in the masseter sNA(n) produces accelerations in the horizontal and vertical directions of the jaw-tongue structure on the sagittal plane [acx(n) and acy(n)] which may be directly estimated from the 3D accelerometer. The dynamic action on this structure changes the configuration of the ONPT, and its filtering function FONPT(z). The radiated speech signal sr(n) is the result of filtering the glottal excitation ug(n) by the ONPT. The inverse filtering of speech reconstructs the inverse transfer function HLP(z), which is used to determine the first two formants F1(n) and F2(n). The dynamic behavior of the first two formants is used to indirectly estimate the correlates of the horizontal and vertical accelerations in the sagittal plane afx(n) and afy(n). Cross-correlation between dynamic and acoustic estimations of acceleration and force from sEMG measurements is used to optimally align formants and accelerations. Linear regression between dynamic and acoustic variables is used to estimate the model weights.

The methodology used is based on linear regression and cross-correlation on the mentioned signals and estimates, as represented in Figure 4.

The systemic approach is intended to establish the strength of the relationships in the cause-effect chain expressed by the following links: Neuromotor Activity → Masseter sEMG → Vertical Force → Vertical Position → {Δ*F*_1_, Δ*F*_2_}. In this problem s_*NA*_ is the ground truth (not directly observable), s_*EMG*_ is its observable correlate, and *a*_*cx*_ and *a*_*cy*_ are the observable dynamic correlates. The estimates of the acoustic accelerations *a*_*fx*_ and *a*_*fy*_ help in establishing a validation for the indirect estimation of kinematic variables directly from acoustics. These kinematic variables would allow estimating the neuromotor activity directly from acoustics using speech recordings from remote devices (Palacios et al., 2020).

### Materials

Eight volunteering speakers (four males and four females) were recruited among PD participants from the APARKAM association of Alcorcón and Leganés, two cities in the southwest of the community of Madrid, Spain. The inclusion criteria were non having had a diagnosis of any laryngeal pathology, being in H&Y stage 2, and non-smokers. Another set of eight participants of both genders with not known laryngeal or neurological pathologies in a similar age range were selected to serve as controls. Their biometrical description is given in Table 1.

**TABLE 1:** Participants’ biometrical data.

**Participant’s code**	**Gender**	**Age**	**Condition**	**H&Y**
CFa	F	67	C	
CFb	F	62	C	
CFc	F	66	C	
CFd	F	67	C	
CMa	M	69	C	
CMb	M	71	C	
CMc	M	68	C	
CMd	M	69	C	
PFa	F	73	P	2
PFb	F	66	P	2
PFc	F	65	P	2
PFd	F	70	P	2
PMa	M	69	P	2
PMb	M	73	P	2
PMc	M	72	P	2
PMd	M	69	P	2

**FIGURE 5:**
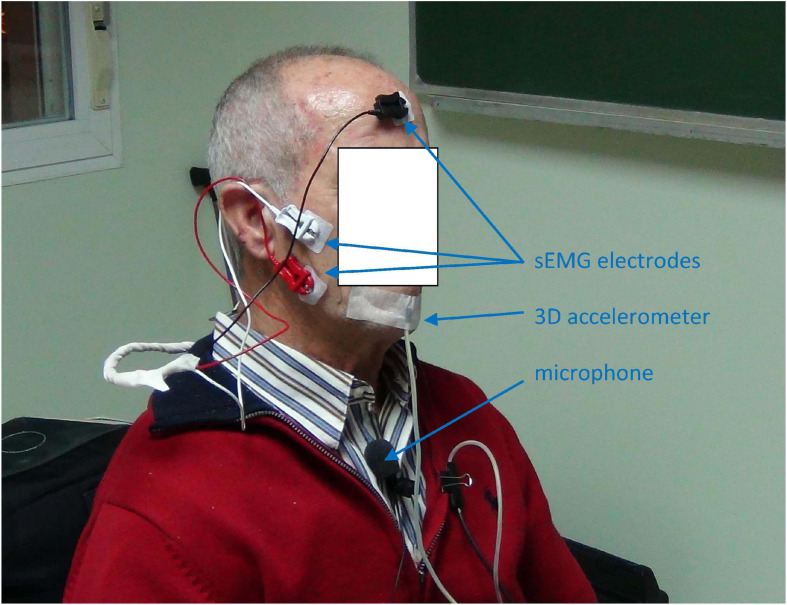
Data recording from a normative male participant: a) the sEMG on the masseter muscle is taken by white and red contact electrodes on the masseter and a reference electrode (black) on the forefront; b) the 3D acceleration is acquired by a 3D accelerometer attached to the chin; the speech signal by a clip wireless cardioid microphone on the chest at 25 cm from the mouth. Jaw-fixed accelerometers are to be used only during signal recording to establish and validate a hands-off acoustic to kinematic projection model, to ultimate use only acoustic signals for at-home monitoring, producing indirect estimations of the neuromotor kinematic characteristics of the participant using the projection model resulting from the study. The participant consented the publication of this anonymized picture.

The study was approved by the Ethical Committee of Universidad Politécnica de Madrid, and the participants signed an informed consent. The experimentation protocols were aligned with the Declaration of Helsinki. The data recording protocol consisted in the synchronous and simultaneous recording of voice, 3D accelerometry and sEMG as shown in Figure 5.

The speech samples were recorded at 50 kHz with16 bits resolution on a MOTU Traveler sound card by a wireless link, using a clip cardioid microphone (Audio Technica) at the speech therapist studio. No special soundproofing room was required due to the short distance to the microphone and its high directionality. The samples from the sEMG and the 3Dacc were recorded at 2.5 kHz. The five signal channels were acquired and digitized by a Biopac M150.

## Results

**FIGURE 6:**
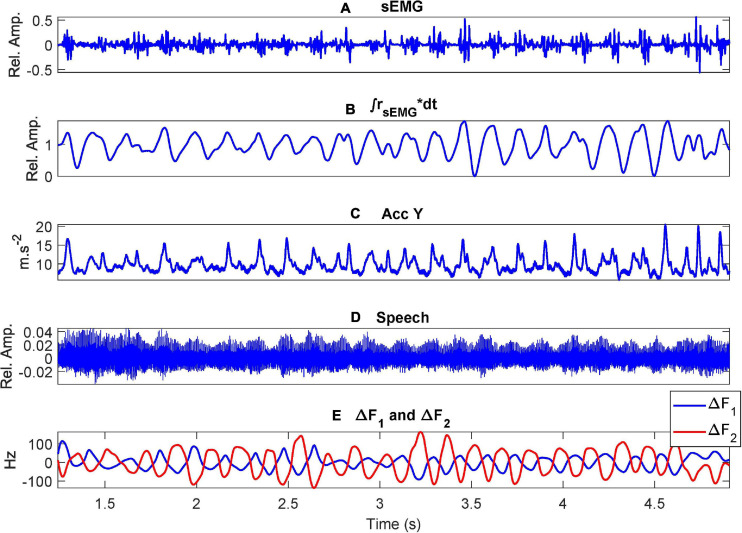
Example recordings when uttering the repetition of […aI…] by a control participant (CMa): **(A)** the sEMG on the masseter muscle; **(B)** the ECMF; **(C)** acceleration on the vertical axis (sagittal plane); **(D)** speech signal; **(E)** first two formant oscillations (unbiased). Where (*) stands for the multiplication operation.

The methodology described refers to concurrent recording of speech, sEMG and 3D accelerometry. The results presented here refer to the use of rotated vertical accelerometry only with respect to the first and second formant oscillations. As an example, the corresponding records for the male participant CM_*a*_ when executing the diadochokinetic exercise of repeating [..*aI*..] are shown in Figure 6.

**FIGURE 7:**
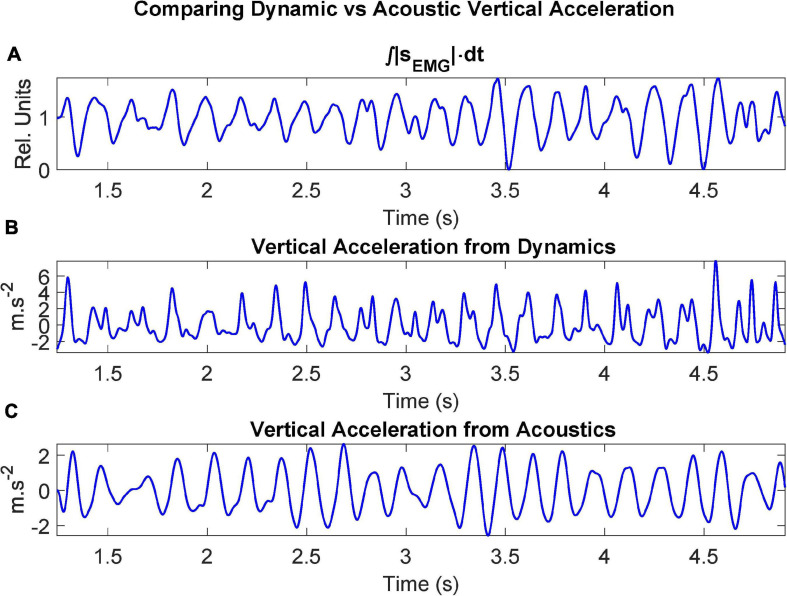
Dynamic signals after processing recordings from the control participant CMa when uttering the repetition of […aI…]: **(A)** ECMF (proportional to the force exerted by the muscle); **(B)** acceleration on the vertical axis after rotation (sagittal plane); **(C)** vertical acceleration estimated from the first two unbiased formants.

The ECMF, the rotated vertical acceleration (dynamic) and the vertical acceleration estimated from the first two formants (acoustic) are shown in detail in Figure 7.

**FIGURE 8:**
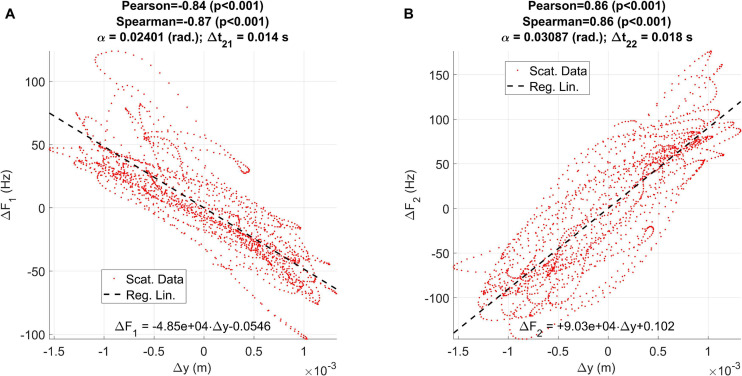
Regression plots between the vertical displacement estimated from accelerometry (Δy) and the first two formant unbiased oscillations: **(A)** relative to ΔF1; **(B)** relative to ΔF2.

The relationship between the vertical displacement Δ*y* and the formant oscillations Δ*F*_1_ and Δ*F*_2_ may be studied using linear regression on the respective signals. The results are presented in Figure 8.

**FIGURE 9:**
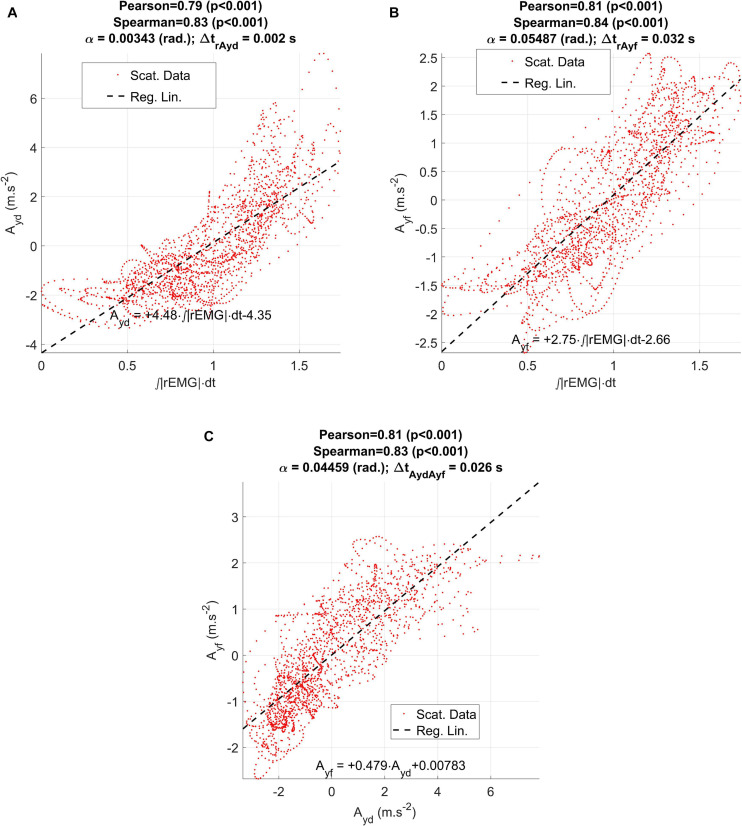
Regression results between the ECMF and the estimated accelerations (dynamical and acoustical) from the participant CMa: **(A)** regression plot between the vertical dynamic acceleration (Ayd) and the ECMF; **(B)** regression plot between the vertical acoustic acceleration (Ayf) and the ECMF; **(C)** regression between the vertical dynamic and acoustic accelerations.

Other aspects of interest are the relationships between the ECMF and the vertical rotated accelerations measured by the accelerometer (dynamic) and estimated from formants (acoustic). As the relationship between these two variables is also of interest for the study the results are presented in Figure 9.

The results of similar evaluations for each participant in the study are given in Table 2 for comparative purposes.

**TABLE 2:** Linear regression results.

**Participants**	**CFa**	**CFb**	**CFc**	**CFd**	**CMa**	**CMb**	**CMc**	**CMd**	**PFa**	**PFb**	**PFc**	**PFd**	**PMa**	**PMb**	**PMc**	**PMd**
*r*_21_	−0.9	−0.77	−0.79	−0.88	−0.84	−0.94	−0.92	−0.91	−0.93	−0.64	−0.85	−0.9	−0.86	−0.93	−0.81	−0.91
*a*_21_ (x10^3^)	−55.5	−58.1	−76.1	−55	−44.8	−78.4	−94.8	−67.5	−205	−74.1	−95.9	−115	−145	−289	−185	−159
*r*_22_	0.94	0.86	0.76	0.85	0.86	0.88	0.87	0.86	0.84	0.53	0.93	0.84	0.81	0.89	0.65	0.83
*a*_22_ (x10^3^)	110	152	135	43.1	86.1	62.0	99.6	71.5	258	119	173	151	173	267	153	181
*r*_*sd*_	0.7	0.78	0.83	0.66	0.79	0.58	0.53	0.71	0.74	0.78	0.81	0.86	0.6	0.49	0.63	0.84
*w*_*sd*_	36.9	8.81	5.01	8.84	4.48	9.24	95.8	35.0	3.94	5.52	30.9	7.27	32.2	26.4	14.2	15.5
*r*_*sf*_	0.72	0.8	0.79	0.69	0.81	0.53	0.59	0.74	0.84	0.79	0.82	0.76	0.67	−0.41	0.68	0.83
*w*_*sf*_	20.4	5.05	3.4	4.9	2.77	4.06	44.9	15.9	0.75	0.96	14.1	3.67	8.15	−14.4	4.23	14.4
*r*_*df*_	0.92	0.87	0.82	0.87	0.81	0.95	0.79	0.93	0.87	0.77	0.91	0.91	0.81	0.91	0.78	0.93
*w*_*df*_	0.50	0.49	0.58	0.46	0.49	0.46	0.40	0.38	0.15	0.13	0.41	0.52	0.18	0.59	0.22	0.87

**r_21_ and r_22_: Pearson’s correlation coefficients between vertical displacement and the first two formants; a_21_ and a_22_: projection weights defined in expression* (1)*; r_*sd*_, and r_*sf*_: Pearson’s correlation coefficients between vertical accelerations estimated from dynamic and acoustic signals and the force estimated from the sEMG; r_*df*_: Pearson’s correlation coefficient between the vertical accelerations estimated from dynamic and acoustic signals; w_*sd*_, w_*sf*_: regression slopes between vertical displacement and the dynamic force estimated from the sEMG; w_*df*_: regression slope between the vertical accelerations estimated from dynamic and acoustic signals.**

**FIGURE 10:**
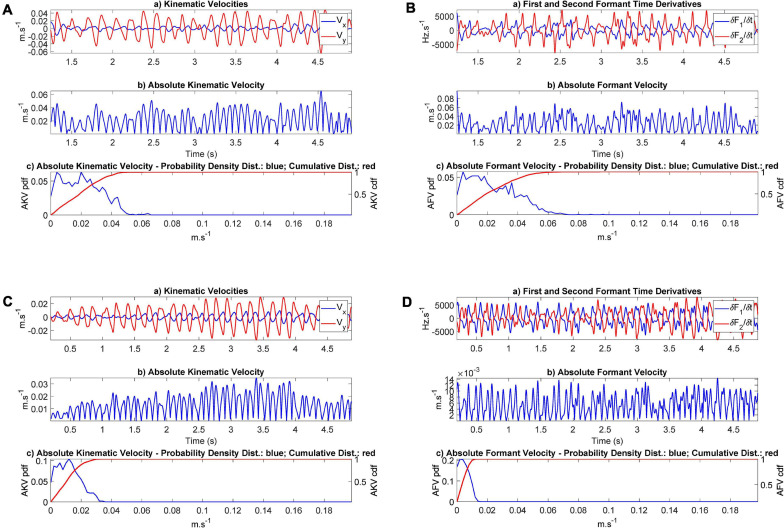
Absolute Kinematic and Formant Velocities (AKV and AFV): **(A)** AKV from participant CMa; **(B)** AFV from participant CMa; **(C)** AKV from participant PMa; **(D)** AFV from the PD participant PMa. In each plot the subplot (a) represents the horizontal and vertical velocities or the corresponding formant derivatives in the time domain. The subplot (b) represents the instantaneous value of the absolute velocity. The subplot (c) gives the density distribution (in blue) and the accumulated distribution (in red). The range of the common scale of velocities goes from 0 to 20 cm.s^− 1^.

As HD is a manifestation of the neuromotor failure in responding to rapid movements, the concepts of absolute velocity defined in (4) and (6) may help in evaluating and quantifying this manifestation. The AKVs and AFVs from a control and a PD participant are represented in Figure 10.

The JSD of each participant with respect to the average distribution of the control participants *p*_*CP*_ defined in (9) to explain individual kinematics with respect to a common reference is given in Table 3.

**TABLE 3:** Results from JSD comparisons on the AKV and AFV distributions.

**Participant’s code**	**JSD/AKV**	**JSD/AFV**
CFa	0.169	0.117
CFb	**0.091**	0.103
CFc	0.110	**0.091**
CFd	0.257	**0.227**
CMa	0.120	0.090
CMb	0.101	0.170
CMc	0.133	0.110
CMd	**0.283**	0.102
PFa	0.326	**0.597**
PFb	0.223	0.436
PFc	0.213	0.336
PFd	**0.124**	0.145
PMa	0.345	0.596
PMb	**0.453**	0.396
PMc	0.423	0.590
PMd	0.332	**0.120**
*p*-value *t*-Test	0.008	0.002
*p*-value KS	0.049	0.010
*p*-value MW	0.010	0.002

**The mid and right columns give the JSDs of each participant with respect to the average AKV and AFV distributions of the control set, p_*PCd*_ and p_*PCf*_, respectively. It may be seen that the JSDs of the PC participants are higher than the ones estimated on the control group. The p-values at the bottom of the table give the results of testing the JSDs from the PD set to the JSDs form the control participants. Values in bold specify the largest and smallest divergences with respect to the control averages.**

The results from the PD cohort are compared with the ones from the control participants using three types of tests: Student’s *t*-test, Kolmogorov-Smirnov (KS) and Mann-Whitney (MW). Previously, the results from each cohort have been tested for normality using the Lilliefors test. The normality hypothesis was not rejected for any of the cohorts (controls and PD). The results of the three tests are given at the bottom of the table. In this case, *t*-test and KS test rejected the null hypothesis of same means under a 0.05 significance level. MW test also rejected the null hypothesis of equal distributions from both cohort results under a 0.05 significance level.

## Discussion

Starting with the recordings from the example presented in Figure 6 it may be seen that the sEMG shows spike-like bursts which correspond to muscle contractions during the articulation movements produced to utter the diadichokinetic exercise (a). The placement of the electrodes is of crucial importance, as well as cleaning the skin with deionized water or a soft de-makeup napkin prior to electrode placement. The ECMF, shown in (b) is well aligned with the rotated vertical acceleration given in (c). Both signals show similar oscillations, which presents narrow peaks, possibly due to the antagonist action of the geniohyoid muscle. The speech envelope (d) shows a larger intensity when the vocal opening is larger (lower sounds when the articulation moves to [*a*]) as the release of acoustic energy is larger during these intervals. It must be mentioned that formants do not arrive to extreme positions on the vowel triangle (→[a] and →[I]), swinging instead between a low and a high vowel, which can be described phonetically as [æ→I→æ]. In (e) the first two formants (in blue and red) show a counter-phase oscillation, which is delay-aligned with the acceleration. Similar plots were also produced from each participant.

Regarding Figure 7, it may be seen that the rotated vertical acceleration as measured by the accelerometer (b) shows a sharper behavior during vertical pull-ups when compared to the ECMF (a), although their positive peaks are well aligned. This may be due to the action of the antagonist muscles (mainly the geniohyoid), which apparently retain the vertical movement up to a point where they suspend the retention and a sharp vertical peak is released. The acoustical vertical acceleration (c) shows a smoother behavior, and although it is also aligned with (a) and (b) a small delay may be observed in the acoustical acceleration, due to the inertial response of the positioning of the jaw, which conditions the establishment of formants within some delay. An added factor to explain this delay is the time required for the acoustic wave to produce the sustained standing waves in the ONPT which are responsible of formants. These delays can be inferred from the regression study shown in Figure 9.

The results of applying linear regression are given in Figure 8, showing that the influence of the vertical displacement affects more the second formant than the first one (an oscillation of 2 mm produces a formant swing of almost 100 Hz on *F*_1_, and about 200 Hz in *F*_2_, peak-to-peak). The effect is reversed on each formant: whereas positive displacements in the vertical axis result in negative displacements on *F*_1_, they induce a rise on *F*_2_, as expected from the description given in Figures 2A,B). The correlation coefficients (Pearson and Spearman) are both large and significant. The delays suggest that the formants react to the vertical displacement with a delay between 14 and 18 ms. This delay is due to the inertial moment of the jaw-tongue structure, and to the time required for the formants to emerge acoustically as standing waves within the acoustical structures, a factor which is also regulated by the quality factors of the resonances, and ultimately on the energy losses caused by viscoelastic factors on the biological tissues, an interesting problem which would deserve further study.

Regarding the regression results shown in Figure 9 between the ECMF and vertical acceleration A_*yd*_ measured from the accelerometer (a: dynamical) and A_*yf*_ from the indirect estimation on the first two formants (b: acoustical) it may be seen that there is a direct regression in both cases, showing large and significant correlation coefficients (Pearson). Nevertheless, the dynamical acceleration shows a small delay with respect to the ECMF (2 ms, which corresponds to a single sample interval at an effective sample frequency of 500 Hz). On its turn, the delay between the acoustical acceleration A_*yf*_ and the ECMF is of 32 ms. When checking the correlation between the dynamical and the acoustical acceleration estimates, a delay of 28 ms is observed in the second one with respect to the first one. The disagreement when considering the delays of both signals in regard to the ECMF is of 4 ms (2 sampling intervals at 500 ms), which could be attributed to small signal misalignment errors.

An exhaustive examination of the results presented in Table 2 shows some interesting observations. For instance, the regression between the vertical displacement and the first formant oscillations (*r*_21_) is the largest in absolute value for CMb, whereas PFb shows the smallest one. No significant difference is appreciated between control and PD participants in this respect. The gain factor *a*_21_ is the largest for PMb, whereas for CMa it is the smallest. In this case, this factor is significantly largest for PD participants than for controls, a fact that would require further study. The regression between the vertical displacement and the second formant oscillations (*r*_22_) is the largest for CFa, and the smallest for PFb. No significant differences were found between control and PD participants. The smallest oscillation gain (*a*_22_) was found for CFd, and the largest for PMb. In this case the estimations from controls and PD participants were also significant. The regression between ECMF and the dynamic vertical acceleration (*r*_*sd*_) showed the largest value for PFc, and the smallest for PMb. No significant differences were observed between the two datasets. The oscillation gain (*w*_*sd*_) produced rather disperse estimations in this case, the largest value is observed for CMc and the lowest for PFa. The regression results between ECMF and the vertical acoustic acceleration (*r*_*sf*_) show the largest value for PFa, and the smallest one for PMb (negative in this case, a counter-intuitive result, possibly due to an inefficient small amplitude recording due to low conductance, this fact requiring a further study). No significant differences were found between the two datasets. The oscillation gain (*w*_*sf*_) showed again a strong dispersion, and the singularity of PMb producing a large and negative value again. This dispersion in the values of *w*_*sd*_ and *w*_*sf*_ may be related with a less efficient recording of the sEMG in some cases, due to skin and fat conductance, as well as electrode placement. On its turn, the correlation between vertical dynamic and acoustic accelerations (*r*_*df*_) showed large values, the difference between the largest (CMb) and the smallest (PFb) being relatively small. No significant differences were observed between the two datasets. Finally, the gain factor between both accelerations (*w*_*df*_) showed the largest and lowest values for PMd and PFb. In this case, a significant difference was observed.

The results presented in Figure 10 show that the distributions corresponding to the control participant extend to higher values of the absolute velocity (5–6 cm.s^–1^) than the values from the PD participant, which barely extend to 3 cm.s^–1^ (AKV) and do not surpass 1.5 cm.s^–1^ (AFV). This may be an indication of hypokinetic behavior, compatible with HD. This same situation was present in all the PD participants tested, as showed in the results found in Table 3. The statistical relevance of this different behavior has been assessed by three statistical tests: *t*-test, Kolmogorov-Smirnov and Mann-Whitney. The rejection of the null hypothesis of equality of distributions may be interpreted in the sense that the sets of JSD from the control and PD participants come from different distributions and are separable based on the value of their respective JSDs. Therefore, the *p*-values of the tests avail a statistical differentiation between controls and patients using JSDs estimated from AKV (dynamic) as well as from AFV (acoustic).

As a general summary it may be said that the correlation studies on sEMG relative to A_*yd*_ and A_*yf*_ presented relevant results both for control and PD participants, which means that the disease in itself is not a differentiation factor regarding the association of myoelectric, dynamic, and acoustic signals, allowing to build chain models to infer the neuromotor activity in the masseter exclusively from acoustic estimates, therefore the assignment from acoustics to neuromotor (the objective of the present work) will work equivalently for both groups, allowing the design and use of an inverse model to project acoustic estimates to neuromotor ones.

Regarding the statistical tests reported on Table 3, a clear different behavior may be appreciated in the PD dataset with respect to the control dataset, as the JSDs of the PD dataset are larger to the average control distributions. The smallest JSDs are found in the control dataset, whereas the largest ones are found in the PD dataset. It may be seen also that the tests reject the null hypothesis of equal distributions between the control and PD participants’ JSDs either estimated from dynamics (AKV) or from acoustics (AFV) under a 0.05 significance level. The differentiation between both groups is of most interest to assess HD by telemonitoring devices recording speech remotely. The validation of this possibility has been already studied using acoustic estimates only (Gómez et al., 2017, 2019a), but a wider study using both methods and ECMF is still pending.

The cross-correlation between the ECMF and the kinematic variables estimated also the delays for signal alignment. The following causes have been determined to explain the delays: the inertial dynamic behavior of the jaw-tongue structure, included in numerical integration (delay); the acoustic kinematic estimation, included in numerical differentiation (anticipation), and the most plausible hypothesis, yet to be tested, which would have to see with the time required for the standing waves in the resonances of the ONPT to attain enough intensity to create an emerging formant, and this process would have to see with the quality factor involved in the equivalent generating resonances, ultimately linked to the losses in the ONPT tissue walls (assumed to be rigid and non-viscous). This hypothesis may be checked tracking the pole positions after each time step (2 ms in the present study).

Apparently, the projection model for PD and control participants does not reveal strong differences as far as regression results show. On the contrary, the differentiation between both datasets is relevant in terms of absolute dynamic and acoustic kinematics. This observation must be taken with some precaution, given the important weaknesses and limitations affecting the study, among them the low number of participants studied, although a steady recruitment process is ongoing facing future studies. A non-deniable factor to be taken into account is the potential HD due to aging in the control group, as a confounding factor regarding neuromotor degeneration by disease. Another important limitation is that the low number of participants per gender did not allow a gender-separated study.

Another possible limiting fact is that all PD participants that have been recruited in the study were H&Y stage 2 and hence the generalization of our findings for different PD stages need to be evaluated in a follow-up study. This was a pragmatic constraint mandated by practical challenges in the recruitment. The study was conducted on members of PD associations in the southwest area of Madrid. These participants had been diagnosed and followed by the public healthcare system and accepted willingly and enthusiastically participating. However, for ethical reasons we could not test them in the OFF state. Therefore, participants in an early H&Y stage 1 did not manifest motor problems associated to speech quite clearly, and most of the recordings were not valid. On the other hand, participants in more advanced PD (above state 2) suffered from other co-morbidities, ad it was challenging to recruit them for the needs of the study. Besides, we observed on the available participants in H&Y stage 3, that the recording session, although not lasting more than 25–30 min for a control participant, was for them an exhausting task. Indeed, most of the time was spent in the preparation of the face skin, fixing electrodes, accelerometer, and microphone. Recordings which would typically last 5–10 min for a control participant, almost doubled in these cases, due to misunderstanding of instructions, repetitions, and participants’ fatigue.

The effects of choosing PD participants in H&Y stage 2 only, could be assessed observing if the estimations of each feature listed in Table 2 differed significantly between HC and PD participants. If both distributions did not differ significantly between HC and PD participants in H&Y 2, possibly it could be expected that distributions from PD participants in H&Y < 2 would not differ significantly as well. Extrapolating this observation to PD participants in H&Y > 2 is not possible considering the data available right now. The comparisons have been done using three statistical tests on the null hypothesis of equal distributions: a parametric *t*-test, and two non-parametric ones, Kolmogorov-Smirnov (KS), and Mann-Whitney (MW) on the correlation and model projection coefficients given in Table 2 (10 features, one per row). We adjusted the significance level of 0.05 per each feature accordingly to Holm-Bonferroni’s correction (Holm, 1979). The adjusted significance levels per feature (in parenthesis) were 0.0050 (1), 0.0056 (2), 0.0063 (3), 0.0071 (4), 0.0083 (5), 0.0100 (6), 0.0125 (7), 0.0167 (8), 0.0250 (9), 0.0500 (10). The sorted *p*-values per row (in parenthesis) were 0.0017 (4), 0.0064 (2), 0.1790 (8), 0.1966 (3), 0.4386 (10), 0.4881 (6), 0.5901 (7), 0.7176 (1), 0.7263 (5), 0.7832 (9). Therefore, the null hypothesis could only be rejected for feature *a*_22_ (4), because its *p*-value (0.0017) was under the lowest significance level (0.0050). Comparing the results from the KS test in a similar way, the null hypothesis could only be rejected for feature *a*_21_ (*p*-value of 0.0014). The results from the MW test rejected the null hypothesis for both *a*_21_ and *a*_22_ (*p*-values of 0.0011), but not for the remnant features. The overall comparison results point to the global acceptance of the complementary hypothesis of equal means, except for the specific features *a*_21_ and *a*_22_.

The effects of the H&Y stage choice could also be assessed observing if the JSD distances considered in Table 3 differed significantly between HC and PD participants. As it may be seen, all the tests reject the null hypothesis of equal distributions of JSD scores respect to a significance level of 0.05, although the case of KS with JSD scores produced from the AKV distribution, is only slightly below the significance level (0.049). This means that probably this test (KS on JSD from AKV) would fail rejecting the null hypothesis for a set of PD participants in stage H&Y < 2. How the other tests would work under the same conditions is less predictable.

The unexplained contribution to the variance in linear regression results (in terms of 1−r^2^, where r is the respective correlation coefficient) by the effects of the ECMF on the vertical acceleration may be due to the action of the geniohyoid and other muscles contributing to the elevation and depression of the jaw-tongue system. The activity of the geniohyoid muscle in depressing the jaw-tongue structure has not been estimated in the present study. The inclusion of sEMG recording electrodes on geniohyoglossus muscle could complement it.

There are several limitations affecting the present study, which have to be mentioned here. First of all, independent jaw-tongue movements are not analyzed, however, it must be stressed that the present study is mainly focused on proposing acoustic features which may be mapped to potential biomarkers of degenerative speech HD of neuromotor etiology, not on a general model in the sense of Ouni and Laprie (2005). Therefore, the proposed model does not cover the whole span of articulatory movements to acoustic features. Instead, reducing the movement space to vertical activity, helps in providing more accurate estimations of neuromotor activity on the masseter. A possible future extension of the study to independent tongue, jaw and lip movements would require monitoring the sEMG activity of other muscles, as the genio-hioglossus or the orbicular muscles. Besides, the model assumed linear relationship between formants and kinematics. Having in mind that the relationship with the biomarkers (acoustic and dynamic AKF and AKV) become non-linear, this is a necessary approach to allow a first conclusive study. Another important limitation is the small size of the dataset included in the study. This limitation will be removed when the pandemic conditions allow for the inclusion of more recordings. Once the projection model is validated using the multi-trait signals (speech, sEMG and 3DAcc) the detection properties of the proposed biomarkers (AKV and AFV) will be tested on larger PD speech databases (Sakar et al., 2013; Orozco-Arroyave et al., 2014), however, as they only contain speech data, we cannot use them for this validation phase, considering the novel direction that this work is proposing. Hopefully we will be able of using the projection model to produce the AKV and AFV biomarkers, to validate the statistical relevance of a speech-based home-monitoring approach on these databases and others recruited by our own using a tele-health platform (see Palacios et al., 2020). Another limiting factor is that PD participants were in a moderate stage of motor activity deterioration (H&Y stage 2). This limitation would require extending the analysis to PD participants across all stages to investigate the changes across the spectrum of the disease, from very early onset (HY stage 1) to very late PD stages.

Another important open issue is how speaker dependency and inter-speaker variability may affect absolute kinematic distributions as disease biomarkers. In this sense, it may be seen from the work of Dromey et al. (2013) that the variability of inter-speaker results for diphthong [aI] is the lowest from all diphthongs these authors have studied per class (control vs. PD participants). Moreover, it may be shown that the projection weights in Table 2, if normalized to the [a_*ij*_] vector norm as â_*ij*_ = a_*ij*_/| a_*ij*_| (see Gómez et al., 2021) do not show relevant inter-speaker variability per class. The way that inter-speaker variability may affect the absolute kinematic distributions (the proposed biomarkers), works different for controls than for PD participants, because these biomarkers are the normalized histogram distributions of the absolute kinematic velocities, and they only express if movement is more distributed toward low absolute velocities (hypokinetic), as is the case for the PD participant plotted in Figures 10C,D or if it spreads along low and high absolute velocities (normokinetic), as is the case for the control participant plotted in Figures 10A,B. Therefore, inter-speaker variability is expressing if the speaker is hypokinetic or not, or to put it in other words, inter-speaker variability between controls and PD participants is an essential role of the proposed biomarkers, provided they are designed to differentiate hypokinetic from normokinetic speech. This separation is availed by the results shown in Table 3, where it may be seen that biomarker distributions (kinematic AKV, as well as acoustic AFV) from PD participants show larger distances (in terms of Jensen-Shannon Divergence) to distributions from control participants. Summarizing, the biomarkers proposed show an inter-speaker variability oriented to differentiate control participants from PD participants, in closed relationship with the second objective of the study as stated in section “Objectives.”

## Conclusion

This study presents a multi-trait evaluation of HD, including speech, 3D accelerometry and sEMG signal acquisition on the masseter. A cross-correlation signal alignment is carriend on these signals. A further regression analysis on the re-aligned acoustic formants, kinematic accelerations and dynamic-related electromyography signals allows the estimation of the projection model parameters. The projected acoustic-kinematic and dynamics-kinematics absolute movement distributions are used as HD biomarkers. An evaluation of these biomarkers in terms of Jensen-Shannon Divergence is conducted on data from control and PD participants, showing the capability of both biomarkers to detect HD in PD speech. The main findings derived from the study are the following:

• A multimodal framework for the assessment of the masseter’s neuromotor activity based on sEMG, 3DAcc and speech, has been used on diadochokinetic exercises from PD and control participants.
• Large cross-correlations between the measured and estimated signals have been observed using linear regression on a small-size data sample of control and PD participants.
• The cross-correlation study did not show relevant differences between control and PD participants.
• The articulation kinematics estimated from 3D accelerometry and from acoustics showed a relevant similarity for all the participants included in the study.
• It was possible to differentiate the speech kinematic behavior of the control and PD participant sets used in this study, using absolute velocities (dynamic and acoustic) of the joint jaw-tongue reference point. Therefore, their amplitude distributions could be used as potential biomarkers of PD speech HD.

The findings of the current study are preliminary, given the limited sample size. We are currently extending our efforts toward the collection of a larger sample size and investigating how well the presented findings generalize across larger cohorts. The definition of an inverse model to infer neuromotor activity from acoustics is also a future objective. The inclusion of sEMG from other muscles active on the jaw-tongue articulation as the geniohyoid is also foreseen. A study regarding the delay on formant-estimated kinematics based on the non-linear properties of the ONPT is considered as well.

## Data Availability Statement

The raw data supporting the conclusions of this article will be made available by the authors, without undue reservation.

## Ethics Statement

The studies involving human participants were reviewed and approved by the Comité de Ética de la Universidad Politécnica de Madrid. The patients/participants provided their written informed consent to participate in this study. Written informed consent was obtained from the individual(s) for the publication of any potentially identifiable images or data included in this article.

## Author Contributions

AG: manuscript preparation, model conceptual design, and implementation, instrumentation setup, and data recording. PG: research guidelines, manuscript preparation, and model implementation. DP and VR: data recording and manuscript preparation. VN: instrumentation design and setup and data recording. AÁ: data processing. AT: research guidelines and manuscript preparation. All authors contributed to the article and approved the submitted version.

## Conflict of Interest

The authors declare that the research was conducted in the absence of any commercial or financial relationships that could be construed as a potential conflict of interest.
